# Palatal Tuberculosis: A Rare Manifestation of Extrapulmonary Tuberculosis

**DOI:** 10.7759/cureus.96809

**Published:** 2025-11-13

**Authors:** Valter Duarte, João Gama, Gonçalo Silva, Rubén Carvalho, Cristina Valente

**Affiliations:** 1 Internal Medicine, Centro Hospitalar Baixo Vouga, Aveiro, PRT; 2 Department of Anatomical Pathology, Unidade Local de Saúde de Coimbra, Coimbra, PRT; 3 Department of Infectious Diseases, Unidade Local de Saúde de Coimbra, Coimbra, PRT

**Keywords:** extrapulmonary tuberculosis (eptb), immunosuppression, oral cavity lesion, palatal tuberculosis, tuberculosis in immigrants

## Abstract

Tuberculosis (TB) remains a relevant public health concern in Portugal, despite the progress achieved in its control and management over recent years. Extrapulmonary manifestations represent a notable proportion of cases, while oral manifestations are uncommon, particularly those affecting the palate, which are rarely reported. The authors describe a case of a 63-year-old Afghan woman with multiple comorbidities, including poorly controlled diabetes mellitus with end-organ damage and heart failure, who presented with a painless ulcerated lesion on the hard palate with erythematous borders and fibrin coverage. Laboratory evaluation revealed a positive interferon-gamma release assay, and a computed tomography showed oropharyngeal tissue densification and calcified pulmonary micronodules. Despite bacilloscopy being negative, sputum culture was positive for *Mycobacterium tuberculosis* complex. Biopsy revealed non-necrotizing granulomatous inflammation, confirming the diagnosis of palatal TB. Standard anti-TB therapy with rifampicin, isoniazid, pyrazinamide, and ethambutol was initiated, resulting in near-complete resolution of the lesion after 30 days. Unfortunately, treatment was discontinued following ischemic hepatitis and myocardial infarction, and the patient subsequently died of septic shock due to a multidrug-resistant nosocomial infection. Palatal TB is a rare condition that can mimic other oral diseases, raising major diagnostic challenges, especially in immunocompromised patients. It should be considered in persistent oral ulcers, and early diagnosis and treatment are crucial for better outcomes.

## Introduction

The incidence of tuberculosis (TB) has been declining in Portugal over the last 20 years. However, the country continues to have the highest incidence of TB in Western Europe, with 14.2 cases per 100,000 inhabitants, as described in the Tuberculosis Surveillance and Monitoring in Europe 2023 report [1]. This high incidence of TB in Portugal (the fourth highest in the European Union) can be partly explained by increasing immigration rates and the poor socio-economic status of immigrants. The immigrant population has a 4.3-fold higher incidence of TB compared to the native Portuguese population, accounting for 27.4% of the cases in 2020 [2].

TB can be classified as pulmonary or extrapulmonary, with pulmonary TB being the most common (69.7% of cases in 2020). Regarding the extrapulmonary manifestations of tuberculosis, the involvement of the oral cavity is rare and primarily affects the tongue and the gums (0.2-1.5%) [3]. Involvement of the palate is exceptionally rare and has only been described in a few case reports [4,5]. The numerous conditions manifesting as ulcerated oral lesions pose challenges for the identification of TB and may delay treatment initiation [5].

## Case presentation

This case report describes a 63-year-old woman born in Afghanistan, who had refugee status in Portugal for the previous five months. She had a history of poorly controlled comorbidities, including dyslipidemia, obesity, non-insulin-dependent diabetes mellitus with end-organ damage - diabetic retinopathy and peripheral arterial disease requiring a right transtibial amputation 10 years earlier, and heart failure with reduced ventricular function and atrial flutter anticoagulated with edoxaban.

She was referred for consultation due to a painless ulcerated palatal lesion of uncertain duration. She reported asthenia, a 5% loss of body weight, and night sweats in the previous three months. She denied experiencing episodes of red eye, skin abnormalities, arthralgia, sicca, or respiratory, gastrointestinal, or genitourinary symptoms. She had no prior history of a TB diagnosis or treatment, and no family history of autoimmune diseases or neoplasms.

**Figure 1:**
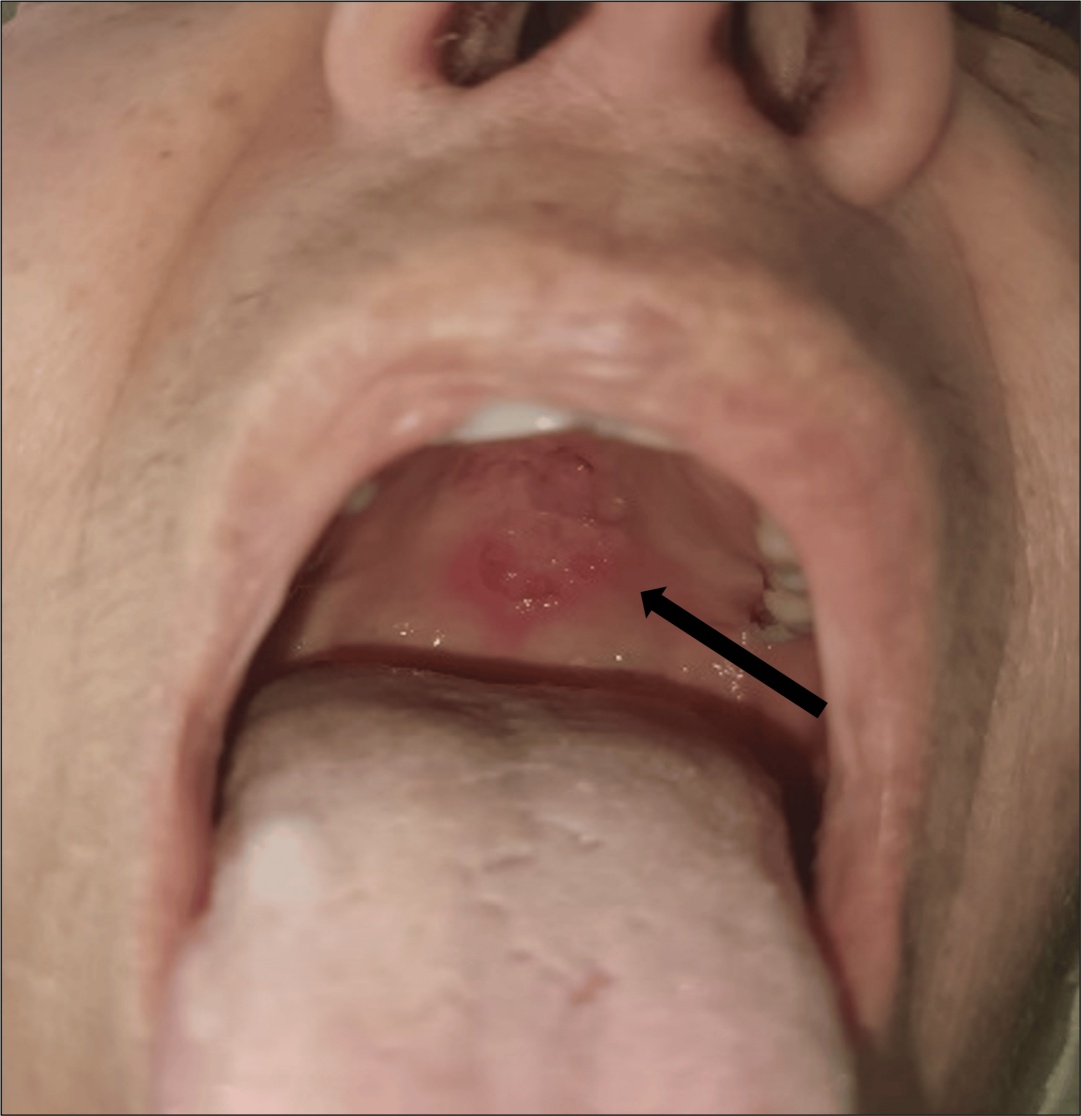
Ulcerated plaque located on the midline of the hard palate (black arrow).

The objective examination revealed an ulcerated palatal lesion with well-defined erythematous borders and fibrin (measuring 8 × 8cm) (Figure 1), with no other oropharyngeal conditions. There were no auscultatory abnormalities, and no adenopathy, skin lesions, or organomegaly were noted.

**Figure 2:**
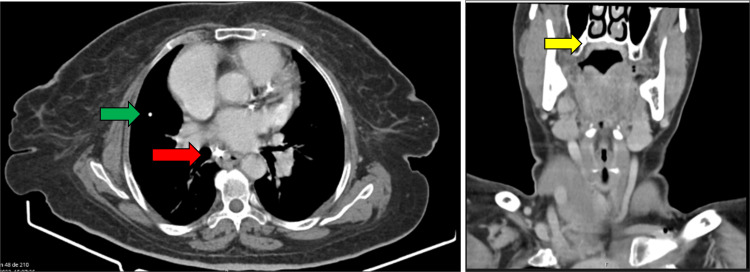
A) Thoracic tomography – Calcified right pulmonary nodule (green arrow) and right hilar lymphadenopathy (red arrow). B) Cervical tomography - Oropharyngeal and palate tissues densification with calcification foci and no bone infiltration (yellow arrow).

Laboratory tests showed poor glycemic control (HbA1c 9.0%) and a positive interferon-gamma release assay. She had acquired immunity for hepatitis A and B viruses, with other serologies (HCV, HEV, HIV, treponemal test) and autoimmunity markers testing negative. A CT scan showed a densification of the oropharyngeal tissues with fine foci of calcification but no bone infiltration, a slight pericardial effusion, a calcified right pulmonary micronodule described as a probable granuloma, and an ipsilateral hilar lymphadenopathy (Figure 2).

**Figure 3:**
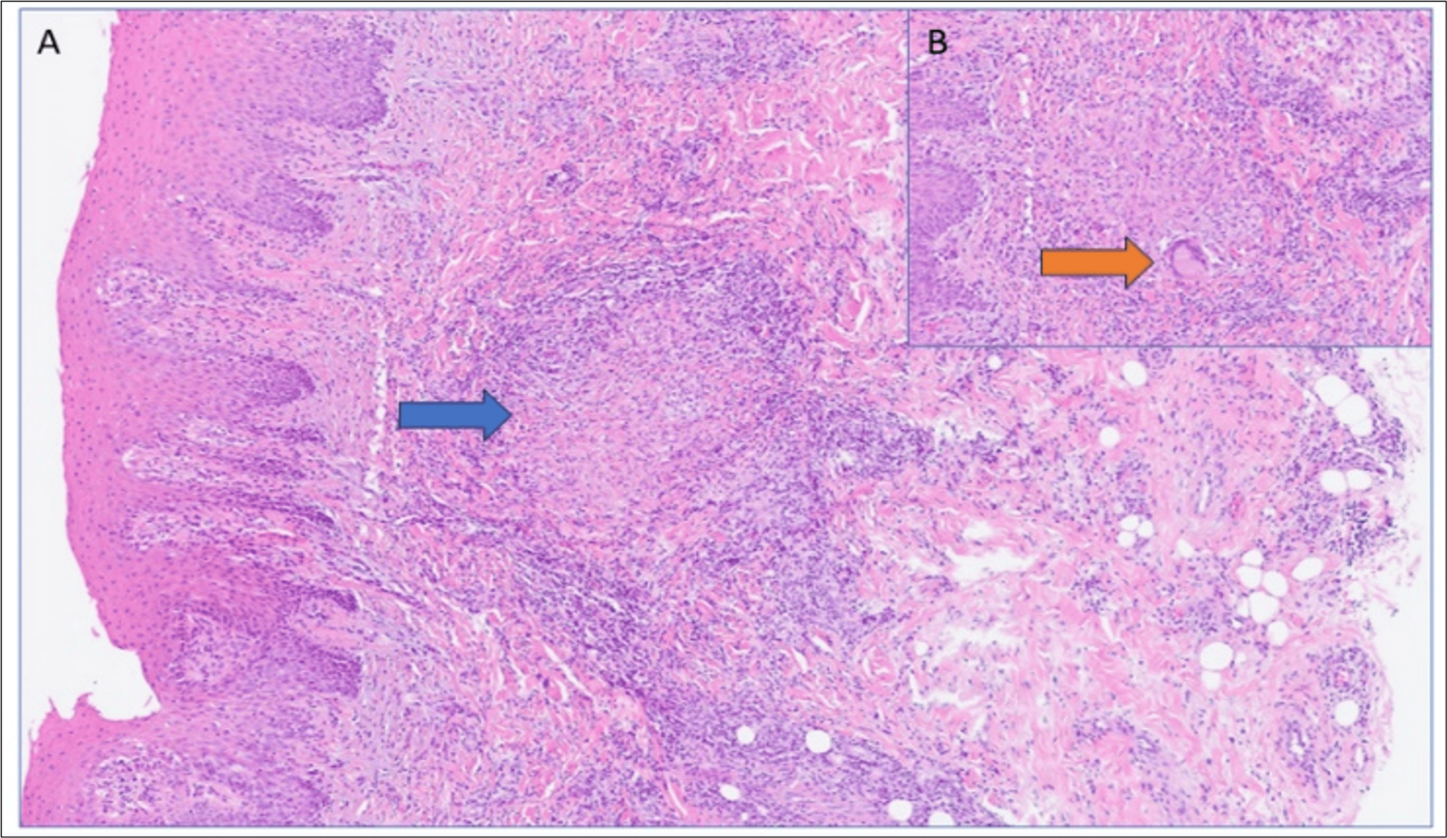
A) Oral tissue biopsy – In the lamina propria, a non-necrotizing granuloma is seen (blue arrow). Hematoxylin-Eosin, 4×. B) Non-necrotizing granuloma with multinucleated Langerhans cell (orange arrow), Hematoxylin-Eosin 10×

The bacilloscopy was negative, but the sputum culture was positive for *Mycobacterium tuberculosis* complex. The remaining cultures for mycobacteria (blood and urine) were negative. A biopsy of the palatal lesion showed evidence of non-necrotizing epithelioid granulomas with a mixed inflammatory infiltrate and multinucleated giant cells (Figure 3). The histological specimen was not cultured.

Based on the biopsy findings and the positive sputum culture, a diagnosis of palatal TB was made, and treatment with rifampicin, isoniazid, ethambutol, and pyrazinamide was initiated. Resistance tests showed susceptibility to all antibacterial agents. After 30 days of treatment, there was an almost complete resolution of the lesion.

Despite the initial promising response, treatment had to be interrupted due to the development of ischemic hepatitis and an acute myocardial infarction. Due to her existing comorbidities and status, conservative treatment was preferred. Before the reintroduction of the antibacterial agents, the patient developed septic shock due to a multidrug-resistant hospital-acquired infection and passed away.

## Discussion

This case highlights the importance of considering TB in the differential diagnosis of ulcerated palatal lesions, particularly in patients with risk factors such as immunosuppressive conditions and immigration from high-incidence regions. The increasing immigration of individuals living in precarious socio-economic conditions, combined with underlying health conditions such as diabetes mellitus with end-organ damage, significantly elevates the risk of TB and extrapulmonary TB. In our patient, diabetes mellitus with end-organ damage was identified as the main predisposing factor for extrapulmonary TB development.

Oral cavity TB can present as either primary, through direct inoculation of *M. tuberculosis*, or secondary, via hematogenous or lymphatic spread. Palatal TB is exceedingly rare, with lesions typically manifesting as granulomas, ulcerations, or perforations, predominantly affecting the hard palate. Given its rarity, the differential diagnosis is broad, encompassing infectious diseases (e.g., candidiasis, herpes simplex virus, leprosy, leishmaniasis), inflammatory conditions (e.g., systemic lupus erythematosus, Wegener’s granulomatosis, sarcoidosis), and malignancies such as squamous cell carcinoma.

Bacilli isolation in sputum and histopathological evidence of palatal granulomas are highly suggestive of palatal TB, and empiric treatment should be initiated. However, a definitive diagnosis of palatal TB relies on the isolation of *M. tuberculosis* from tissue cultures. In this case, a second biopsy was deemed unnecessary due to multiple supporting findings: the positive sputum culture, the presence of granulomas on palatal biopsy, the detection of calcified pulmonary nodules suggestive of prior pulmonary TB, and the patient's origin from a region with a high TB incidence. These factors strongly supported the diagnosis, minimizing the need for additional invasive testing.

Standard treatment for palatal TB consists of a two-month regimen of rifampicin, isoniazid, ethambutol, and pyrazinamide, followed by four months of rifampicin and isoniazid. Although our patient initially showed significant clinical improvement, treatment discontinuation was necessary due to severe adverse effects, ultimately leading to a fatal outcome. This underscores the challenges of TB management in patients with complex comorbidities and highlights the need for close monitoring of treatment tolerance, particularly in vulnerable populations.

## Conclusions

TB should remain a diagnostic consideration in persistent ulcerated oral lesions, especially in high-risk populations. Early recognition and timely initiation of therapy are essential for improving outcomes. Additionally, this case underscores the need for heightened awareness and targeted screening strategies in immigrant populations with socio-economic vulnerabilities and comorbid conditions that predispose them to TB reactivation.

## References

[1] (2025). European Centre for Disease Prevention and Control, WHO Regional Office for Europe: Tuberculosis Surveillance and Monitoring in Europe. Europe.

[2] Relatório de vigilância e monitorização da tuberculose em Portugal (2025). Relatório de vigilância e monitorização da tuberculose em Portugal. http://chrome-extension://efaidnbmnnnibpcajpcglclefindmkaj/https://www.sppneumologia.pt/uploads/subcanais2_conteudos_ficheiros/relat%C3%A3%C2%B3rio-tuberculose_dgs2021.pdf.

[3] Jain P, Jain I (2014). Oral manifestations of tuberculosis: Step towards early diagnosis. J Clin Diagn Res.

[4] Solanki S, Gadre UC, Solanki M, Kaur R (2015). Tuberculosis of palate. Lung India.

[5] Krawiecka E, Szponar E (2015). Tuberculosis of the oral cavity: An uncommon but still a live issue. Postepy Dermatol Alergol.

